# Development of a genetic sexing strain in *Bactrocera carambolae *(Diptera: Tephritidae) by introgression of sex sorting components from *B. dorsalis*, Salaya1 strain

**DOI:** 10.1186/1471-2156-15-S2-S2

**Published:** 2014-12-01

**Authors:** Siriwan Isasawin, Nidchaya Aketarawong, Sittiwat Lertsiri, Sujinda Thanaphum

**Affiliations:** 1 Department of Biotechnology, Faculty of Science, Mahidol University, Rama VI Road, Bangkok 10400, Thailand

**Keywords:** Bactrocera carambolae, Bactrocera dorsalis, genetic sexing strain, Sterile Insect Technique, area-wide integrated pest management, interspecific mating, introgression, microsatellite DNA, male pheromone

## Abstract

**Background:**

The carambola fruit fly, *Bactrocera carambolae *Drew & Hancock is a high profile key pest that is widely distributed in the southwestern ASEAN region. In addition, it has trans-continentally invaded Suriname, where it has been expanding east and southward since 1975. This fruit fly belongs to *Bactrocera dorsalis *species complex. The development and application of a genetic sexing strain (Salaya1) of *B. dorsalis *sensu stricto (s.s.) (Hendel) for the sterile insect technique (SIT) has improved the fruit fly control. However, matings between *B. dorsalis *s.s. and *B. carambolae *are incompatible, which hinder the application of the Salaya1 strain to control the carambola fruit fly. To solve this problem, we introduced genetic sexing components from the Salaya1 strain into the *B. carambolae *genome by interspecific hybridization.

**Results:**

Morphological characteristics, mating competitiveness, male pheromone profiles, and genetic relationships revealed consistencies that helped to distinguish Salaya1 and *B. carambolae *strains. A Y-autosome translocation linking the dominant wild-type allele of white pupae gene and a free autosome carrying a recessive white pupae homologue from the Salaya1 strain were introgressed into the gene pool of *B. carambolae*. A panel of Y-pseudo-linked microsatellite loci of the Salaya1 strain served as markers for the introgression experiments. This resulted in a newly derived genetic sexing strain called Salaya5, with morphological characteristics corresponding to *B. carambolae*. The rectal gland pheromone profile of Salaya5 males also contained a distinctive component of *B. carambolae*. Microsatellite DNA analyses confirmed the close genetic relationships between the Salaya5 strain and wild *B. carambolae *populations. Further experiments showed that the sterile males of Salaya5 can compete with wild males for mating with wild females in field cage conditions.

**Conclusions:**

Introgression of sex sorting components from the Salaya1 strain to a closely related *B. carambolae *strain generated a new genetic sexing strain, Salaya5. Morphology-based taxonomic characteristics, distinctive pheromone components, microsatellite DNA markers, genetic relationships, and mating competitiveness provided parental baseline data and validation tools for the new strain. The Salaya5 strain shows a close similarity with those features in the wild *B. carambolae *strain. In addition, mating competitiveness tests suggested that Salaya5 has a potential to be used in *B. carambolae *SIT programs based on male-only releases.

## Background

Many tephritid fruit flies are serious economic pests with regard to fruits and vegetables. Their infestations, outbreaks, and invasions cause severe damage to crop yields, fruit quality, and international marketing potential [1]. The carambola fruit fly, *Bactrocera carambolae *Drew & Hancock is widely distributed and roughly indigenous to western side of the Indo-Australian Archipelago (demarcated based on fauna bio-geographical survey by Wallace's and Huxley's lines) which includes Peninsular Thailand, Malaysia, and Western Indonesia [2-4]. It infests at least 26 species of host worldwide; most of these fruits are commercial (e.g., star fruit, mango, rose apple, jackfruit, breadfruit, orange) [3,5]. It is regarded as a high profile key pest because it was first trans-continentally discovered in Suriname in 1975 [6] and later described as *Bactrocera carambolae *by Drew & Hancock 1994 [7]. Pest risk analyses have suggested that infested fruit transportation has been mediated by airplane flights for tourism and trade between Indonesia and Suriname [3,5]. The flies had subsequently invaded Northern Brazil (Oiapoque, Amapá) in 1996 from French Guiana [5]. *B. carambolae *is one species in the process of being eradicated from the region north of Brazil [8].

The carambola fruit fly also belongs to a large *B. dorsalis *complex which composes of nearly 100 species. Four members of this species, such as *B. dorsalis *s.s. (Hendel), *B.papayae *Drew & Hancock, *B. philippinensis *Drew & Hancock, *B. carambolae *Drew & Hancock are serious agriculture pests which are wildly endemic in Southeast Asia [7,9,10]. These pests share an overlapping host range and geographical distribution. The identification of these species relies predominantly on morphological characters. However, molecular systematic [11,12], population genetic [13], cytogenetic [14], chemotaxonomy [15,16], host preferences and geographical coordination [17], and mating test studies [18] have been applied to resolve the species limit and description for each member in this complex. With the support of these various lines of evidence, the species classification has been harmonized. For example, *B papayae *has recently been synonymised with *B. philippinensis *[10,19]. On the other hand, *B. carambolae *appears to be a separate species based on mating incompatibility [18], male pheromone profile [20], as well as molecular systematic [11,21]. Nevertheless, there is evidence that both natural and laboratory interspecific hybridization between *B. papayae *and *B. carambolae *has occasionally occurred [11,20].

As the world trade of agricultural commodities is rapidly growing, managing the risks of introducing exotic insects and chemical-insecticide-contaminated fruits into new areas is imperative. Several area-wide integrated pest management (AW-IPM) programs have successfully mitigated the fruit fly problems [22]. In such programs, the entire fruit fly population in a delimited locality is considered. The area layout usually constitutes a large core area and surrounding buffer zone. Within this immediate area, several fruit fly population suppression activities such as surveillance for baseline data and planning, fruit orchard phytosanitation, natural enemy augmentation, male annihilation trapping (MAT), and field monitoring are implemented [22]. The deployment of those conventional control methods is usually efficient with high fruit fly population densities. Further fruit fly population reduction, prevention, and/or eradication would require a complementary sterile insect technique (SIT) that is inverse density dependent [22]. This is because the sterile flies are mobile control agents that seek mates even in areas that are not reachable by other control agents and are beyond the immediate area of application [23].

SIT implementation is one of genetic control strategies that is based on the presence of dominant lethal genetic factors (genetically damaged sperms) in irradiated insects for reducing target populations. The massively field-released sterile males competitively mate with the fertile females to generate nonviable eggs [22]. This birth control practice reduces the population density in the next generation. SIT is target-species specific, environmental friendly, and compatible with organic farming [24]. Traditional SIT program, however, relies on the bisexual release of sterile insects. This poses a few major drawbacks. For example, the sterile females divert the sterile males from mating to wild females and generate stinging damage to the fruits from their ovipositors. The cost of the female mass-rearing production and releasing logistics is also substantial. Desirable male-only releases have been successfully done due to development of genetic sexing strains (GSSs) [25,26].

Genetic sexing strains must facilitate sex-sorting schemes (sexual separation, selection, sexual transformation) based on any stable sex-specific phenotypes of sterile insects for the mass-rearing industry. An example of strain development that has had a significant global impact on SIT programs is the application of GSSs against the Mediterranean fruit fly (medfly) in AW-IPM programs [27]. These types of fruit fly GSSs were developed using the classical genetics approach. They were based on two principle components: a selectable recessive marker that can be used for sorting or killing of females and a Y-autosome translocation that links the dominant wild-type alleles of this marker to the male sex [25,26]. In the resulting strains, the males were heterozygous displaying a wild-type phenotype, while the females were homozygous for the selectable marker, and therefore mutant, and could be distinguished from males. In this medfly case, GSSs also carried the temperature sensitive lethal marker that allowed eliminating females during embryogenesis [28]. During the last few decades, the application of this classical genetic approach had been transferred to only a handful of fruit fly species [29-31] (such as the Salaya1 GSS of *B. dorsalis *which has been validated in a pilot IPM program [30]) due to somewhat difficulty of the method. Ones of these disadvantages are the genetic instability and semi-sterility of the strains generally results from a relatively high frequency of translocation breakdown and aneuploidy, respectively. Moreover, suitable Y-chromosome translocations from the successful medfly genetic sexing strains have not yet been transferred or transmitted to any different species by the classical genetic means. Therefore, novel Y-autosome translocation elements that are as suitable as those in medfly (Vienna8 GSS) [27] or *B. dorsalis *(Salaya1 GSS) [30] would need to be obtained every time for every new pest species, unless a means for introgression is possible, developed, and evaluated.

Alternatively, the recent progress of transgenesis allowed development of sexing technologies which have a better potential to be transferred to a wide range of fruit fly species in the genus of *Ceratitis, Anastrepha*, and *Bactrocera *[26,32-36]. In this case, genetic sexing tools are universally based on a gene transfer system and a sex-specific expression of genes facilitating sex sorting. The gene delivery system usually relies on transposon-based germ line transformation, sometimes deploys site-specific recombinase-based gene targeting, and usually uses fluorescent protein-based markers for the selection of transformants [32,33]. However, the application of relatively recent transgenic-based GSSs for SIT calls for different technical considerations of engineering specific traits, GMO (genetically-modified-organism)-based biosafety regulatory framework, and a developing track record to gain public acceptance [26,37]. Nevertheless, successful but limited open field trials were tested for the release of non-tephritid transgenic insects [37].

The idea of using sterile male fruit flies of a closely related species, *B. dorsalis*, (at a mass-rearing facility from Hawaii) to control *B. carambolae *in Suriname originated from McInnis et al (1999) [38]. These authors revealed that the oriental fruit fly males can copulate with carambola fruit flies in outdoor field cages, although very little cross-mating and no definite sperm transfer were observed due to the limited sample size [38]. Consistently, study of mating compatibility of the pest members within the *B. dorsalis *complex showed that only *B. carambolae *demonstrated a relatively high degree of incompatibility with the other species [18]. It was hypothesized that different pheromone compositions [15,16], other courtship signals, and mating locations (demonstrated from the field cage) may be the cause of low interspecific mating compatibility between *B. dorsalis *and *B. carambolae*.

The prezygotic mating isolation phenomenon [39] of the *B. carambolae *has significant implication for the effectiveness of pest management programs using sterile *B. dorsalis *to suppress *B. carambolae*. This is because the sterile males of *B. dorsalis *may not be able to compete in mating with the wild *B. carambolae *females. The direct application of a translocation-based genetic sexing strain (Salaya1) of *B. dorsalis *to control the *B. carambolae *is unlikely to be effective. Nonetheless, actual mating competitiveness testing is required to confirm this supposition. On the other hand, the postzygotic barrier [39] between *B. carambolae *and the other members of the same species complex does not reveal an obvious incompatibility. There is evidence of natural hybridization and interspecific hybrid reveals intermediate characters. Moreover, the F_1_, F_2_, and back cross hybrids are viable and fertile in laboratory conditions [12,20].

For this work, the extant interspecific hybrids between the *B. dorsalis *and *B. carambolae *allows genetic crossing to stably integrate the validated sex sorting components from the Salaya1strain into the gene pool of *B. carambolae*. This can be done by a genetic strategy called introgression. The introgressive hybridization between Salaya1 and *B. carambolae *was followed by multiple backcrossing with the latter species. A new genetic sexing strain, Salaya5, has been developed. The strain characterization was carried out based on morphological-based taxonomic characteristics, distinctive pheromone components, microsatellite DNA, genetic relationships, and mating competitiveness tests. The Salaya5 strain has close similarity to those features of the wild *B. carambolae*. In addition, mating tests suggested that Salaya5 has a potential to be used in *B. carambolae *SIT programs based on male-only releases. This work is a proof of concept for using the introgression approach to develop a classical genetic sexing strain from a closely related species belonging to the same species complex.

## Results and discussion

### Evaluation of parental characters *B. dorsalis *s.s. (Salaya1 strain) and wild *B. carambolae *(Jakarta)

**Figure 1:**
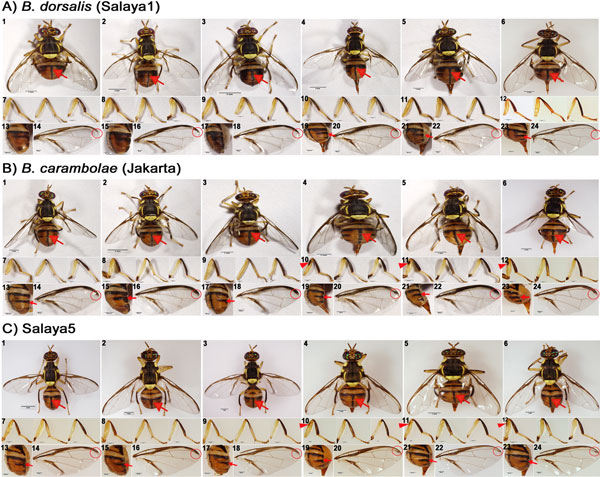
**Morphological characters distinguishing the *B. dorsalis *and *B. carambolae *flies.***B. dorsalis *(Salaya1) (A), *B. carambolae *(Jakarta) (B), and Salaya5 (C); Fruit fly male and female individuals are in sub-figures 1 to 3 and 4 to 6, respectively; the red arrow is pointed at the abdominal terga III-V, the medial longitudinal dark band. Their respective right legs are below in sub-figures 7 to 12, the red triangle is pointing at a subapical dark spot on outer surfaces of fore femora in females. The respective lateral right abdomens (side-view) are also below in sub-figures 13, 15, 17, 19, and 21, respectively; the red arrows are pointed at right antero-lateral corner with a rectangular bar shape in (B) and (C) which are present in *B. carambolae *and Salaya5. Likewise, the respective right wings are below in sub-figures 14, 16, 18, 20, 22, and 24; red open circles indicate wing costal bands expanding at apex of R_4+5 _which are present in *B. carambolae *and Salaya5.

*B. dorsalis and B. carambolae *have several morphological character differences according to Drew and Hancock (1994) [7]. Observation at the abdominal terga III-V reveals that the medial longitudinal dark band is narrow (Figures 1A1 to 1A6) and the anterolateral corners of tergum IV are triangular (Figures 1A1 to 1A6 and Figures 1A13, 1A15, 1A17, 1A19, 1A21, and 1A23) for *B. dorsalis*. However, the medial longitudinal black band is of medium width (Figures 1B1 to 1B6) and the anterolateral corners of tergum IV are large, rectangular, and bar-shaped (Figures 1B1 to 1B6 and Figures 1B13, 1B15, 1B17, 1B19, 1B21, and 1B23) in *B. carambolae*. Observation at the legs (femora) reveals that the femora are entirely fulvous (Figures 1A7 to 1A12) for *B. dorsalis*. The femora of the *B carambolae *are also entirely fulvous (Figures 1B7 to 1B12) but have a subapical dark spot on the outer surfaces of the fore femora, usually in females (Figures 1B10 to 1B12). In addition, the wing costal band observation (Figures 1A14, 1A16, 1A18, 1A20, 1A22, and 1A24) reveals that it is confluent with R_2+3 _and remains narrow and of uniform width to the apex of the wing (occasionally with a slight swelling around the apex of R_4+5_) for *B. dorsalis*. The costal band of *B. carambolae *overlaps with R_2+3_, especially before the apex of this vein, and expands across the apex of R_4+5 _(Figures 1B14, 1B16, 1B18, 1B20, 1B22, and 1B24).

Although the *B. dorsalis *species complex has several noticeable morphological diagnostic characters in adults, discrimination between *B. dorsalis *and *B. carambolae *is sometimes difficult because specimens whose morphological characters are within an intermediate range segregate within a population. These flies with intermediate characters may be natural hybrids in sympatric locales [11]. This work had collected and compared the two species samples from allopatric locations where the present of natural hybrids were highly unlikely. The character comparison is therefore in agreement with most of traditional morphological features [7].

**Figure 2:**
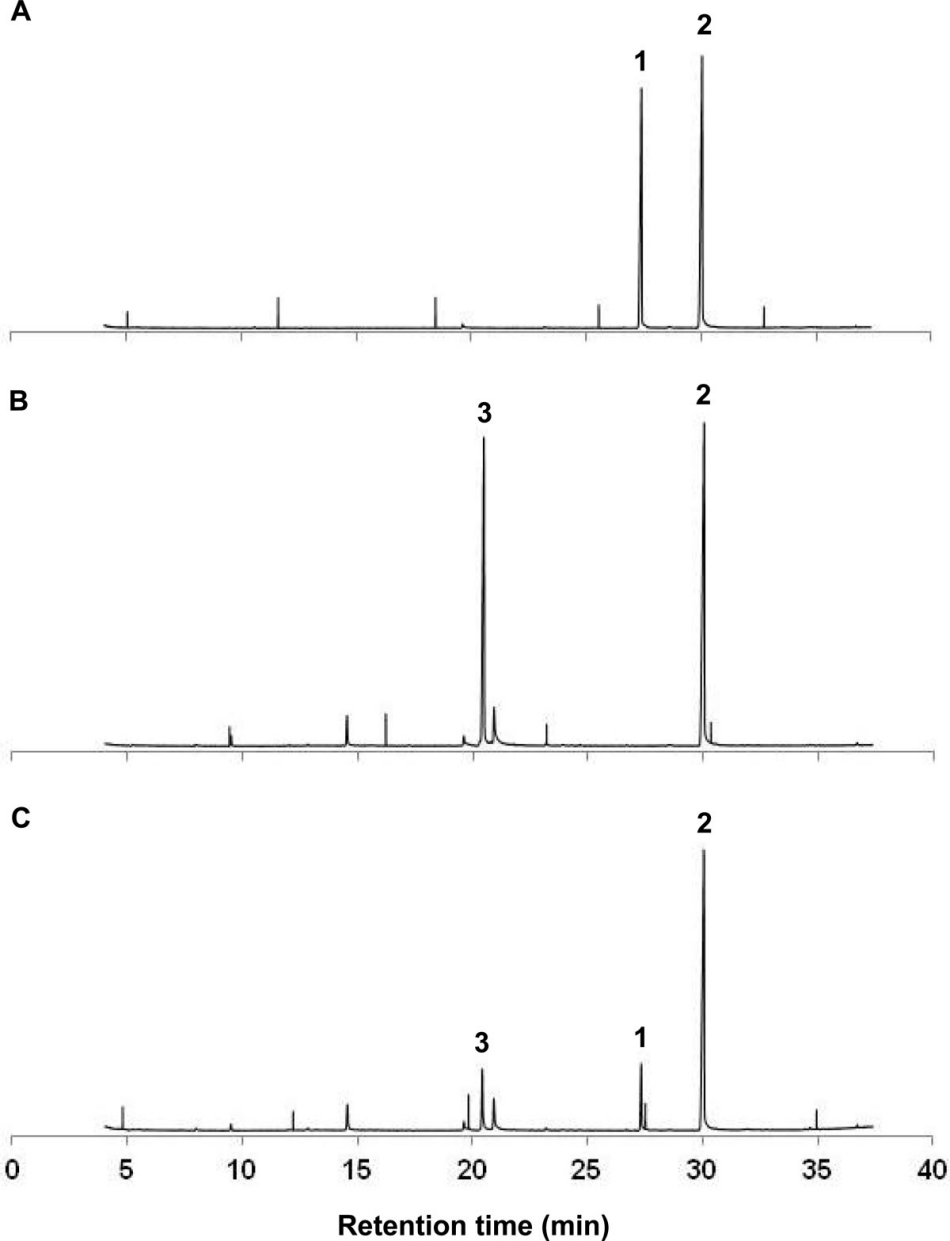
**Gas chromatograms of pheromone profile from male rectal glands of *B. dorsalis *and *B. carambolae***. *B. dorsalis *(Salaya1) (A), *B. carambolae *(Jakarta) (B), and Salaya5 (C), after methyl eugenol consumption, tentatively identified as (1) 4,5-dimethoxy-2-(2-propenyl)phenol, (2) (*E*)-coniferyl alcohol, and (3) 6-oxo-1-nonanol.

The other type of distinctive chemical characters between *B. dorsalis *and *B. carambolae *is the volatile components of the methyl eugenol (ME) fed male rectal glands [15,16]. Each of the rectal glands of the *B. dorsalis *(Salaya1 strain) males after ME consumption contained a distinctive 4, 5-dimethoxy-2-(2-propenyl) phenol (DMP) and non-distinctive (*E*)-coniferyl alcohol (CF), whereas only CF was detected along with a distinctive major endogenous rectal gland component, 6-oxo-1-nonanol (OXO), in that of individual wild *B. carambolae *males (Figures 2A and 2B). These rectal gland male pheromone profiles can consistently differentiate the Salaya1 strain and the *B. carambolae *according to chemotaxonomic references [16,20].

**Figure 3:**
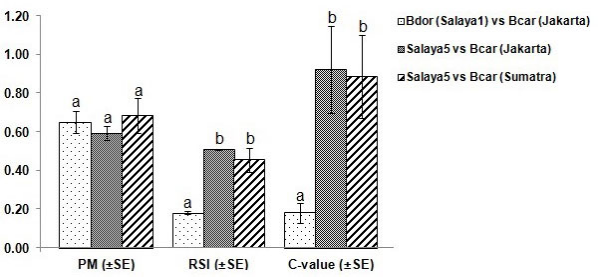
**Field cage mating experiments of *B. dorsalis *(Bdor) and *B. carambolae *(Bcar)**. Salaya1 and Salaya5 are genetic sexing strains were sterilized by irradiation. Wild flies of *B. carambolae *originated from Indonesia (Jakarta and Sumatra). The mating test indices PM, RSI, and C are propensity of mating, relative sterile index, and Fried's competitiveness index, respectively. Different letters represent significant differences (α = 0.05) after *t*-test for equality of means.

The field cage mating tests, in particular the mating competition, between the sterile males of the Salaya1 strain (*B. dorsalis*) and the wild males of *B. carambolae *for the wild females of *B. carambolae *were carried out. This was done when the fruit flies were sexually mature as indicated by a fly mating (PM) value above 60% (Figure 3). The resulting analyses reveal that the relative sterility index (RSI) and the sexual competitiveness (C) are as low as 0.18 (Figure 3). These mating test indices suggest that the wild females preferred to mate with the wild males rather than to the sterile Salaya1 males. This is in agreement with the relatively high degree of mating incompatibility between the *B. dorsalis *and *B. carambolae *[18].

### Introgression

**Figure 4:**
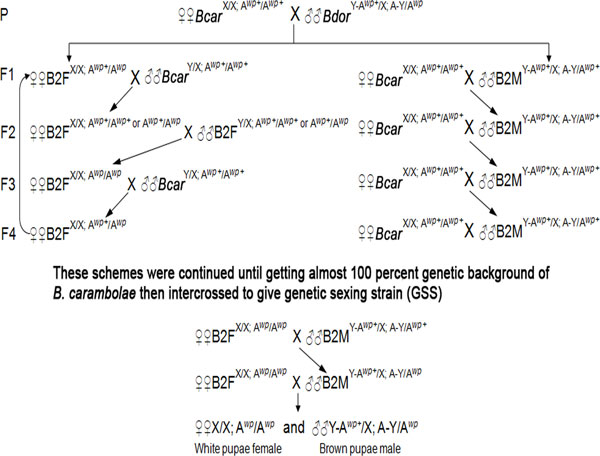
**Introgressive mating scheme for the construction of a genetic sexing of *B. carambolae*, Salaya5**. Bcar, *B. carambolae*; Bdor, *B. dorsalis*; P, parents; F, filial generation and number; B2F, female line; and B2M, male line. *wp^+ ^*and *wp *are wild type and mutant alleles, respectively. A*^wp+ ^*and A*^wp^* refer to the free autosome carrying the wild type and mutant alleles, respectively. Y-A*^wp+ ^*and A-Y denote the two reciprocal components of the Y-autosome translocation.

The resulting F_12 _generation of the B2F line had descended from a repeated loop of the backcross-inbreeding-backcross scheme (i.e. F_1 _to F_3_, F_4 _to F_6_, F_7 _to F_9_, and F_10 _to F_12_; as suggested in the left column of Figure 4). The females (B2F) had, conceptually, a 99.6% genetic background of *B. carambolae*. Likewise, the F_10 _generation of the B2M line (B2M) had, conceptually, a 99.9% genetic background of *B. carambolae*. They were derived from the repeated backcross scheme as shown in the right column of Figure 4. All of the resulting offspring of B2F and B2M were true-breeding for white pupae and brown pupae characters, respectively. The white pupae phenotype in B2F offspring inferred that the white pupae marker (A*^wp^*/A*^wp^*) was completely introduced from the Salaya1 strain. However, the B2M line tentatively carrying the Y-A*^wp+ ^*component still expresses all brown pupae characteristic in both sexes. This is because the A*^wp ^*alleles had not yet been introduced during the introgression process. Therefore, the male progenies of the B2M line (with Y-A*^wp+^*/X; A-Y/A*^wp+ ^*genotype) were crossed with the virgin females of B2F (with A*^wp^*/A*^wp ^*genotype) to generate heterozygote Y-A*^wp+^*/X; A-Y/A*^wp ^*progenies as shown in the middle column of Figure 4. These heterozygote progenies were then backcrossed with the B2F females to reproduce new true breeding brown-white pupae sexual dimorphisms (as shown in the bottom line of Figure 4). This new genetic sexing strain is called Salaya5.

This work is a proof of concept of using forward genetics in the development of genetic sexing strain from a closely related species. Sex specific brown-white pupae genotypes had initially been identified and developed in Salaya1 strain. Subsequently, they were backcrossed according to mating schemes to *B. carambolae*. The progenies were isolated according to individual phenotype which can transfer the genetic sexing components into Salaya5 strain.

### Salaya5 strain characterization

The recognizable morphological diagnostic characteristics of the Salaya5 fruit flies are more similar to *B. carambolae *than to the Salaya1 strain. Observation of abdominal terga III-V in Salaya5 reveals that the medial longitudinal dark bands are not as narrow as those in Salaya1 (Figures 1A1 to 1A6 versus 1C1 to 1C6). The anterolateral corners of tergum IV are larger, with uniformly rectangular shape (Figures 1C13, 1C15, 1C17, 1C19, 1C21 and 1C23), which are similar to *B. carambolae*. The femora are still entirely fulvous, but a subapical dark spot on outer surfaces of the fore femora, usually in females, is present, similar to the *B. carambolae *(Figures 1C10 to 1C12 versus 1B10 to 1B12). Although the wing costal bands do not obviously overlap with R_2+3_, they often expand across the apex of R_4+5 _(Figures 1C20, 1C22, and 1C24) as in *B. carambolae*. These morphological characters suggest that the Salaya5 has similar genetic background of parental *B. carambolae *strain. This argument is based on a fact that many genetic constituents in the genomic background can express non-intermediate morphological characters.

The rectal gland pheromone profile of Salaya5 contains a major distinctive endogenous volatile component (OXO) that belongs to *B. carambolae *(Figure 2B and 2C). After the ME consumption, the rectal glands of the male individuals still contain DMS and CF. Since the presence of OXO is specific to *B. carambolae*, this is also a confirmation that the genetic background of the Salaya5 is becoming *B. carambolae*.

The field cage mating tests were carried out when the fruit flies were sexually mature, as indicated by the PM values in Figure 3. The mating competition between the sterile males of the Salaya5 strain and the wild males of *B. carambolae *against the wild females of *B. carambolae *were tested using wild fruit flies from two remote geographical locations: Jakarta and Sumatra islands. The analyses show that the RSI and C values are approximately 50% and 90%, respectively (Figure 3). These mating indices suggest that the sterile Salaya5 males are not significantly different from the wild males in terms of mating competitiveness.

Five microsatellite loci (*Bd*1, *Bd*9, *Bp*125, *Bp*173, and *Bp*181) were polymorphic in terms of average number of alleles (*n*_a_) and effective number of alleles (*n*_e_): *Bd*1 (*n*_a _= 5.40, *n*_e _= 3.163); *Bd*9 (*n*_a _= 6.60, *n*_e _= 4.248); *Bp*125 (*n*_a _= 3.00, *n*_e _= 1.832); *Bp*173 (*n*_a _= 4.80, *n*_e _= 2.149); *Bp*181 (*n*_a _= 3.40, *n*_e _= 1.944). Deviation from Hardy-Weinberg Expectations (after the sequential Bonferroni correction [40]) was observed in 8 out of 25 populations by locus comparisons. All departures were not concentrated in any populations or any loci. Significant linkage disequilibrium was not detected between a pair of five loci.

For other four Y-pseudo-linked microsatellite loci (*Bd*15, *Bd*42, *Bp*53, and *Bp*73), we consistently observe fixed genotypes or strain identification markers in the Salaya1 strain as established in [30] (Additional file 1). However, the same characters are not detected in the new genetic sexing strain, Salaya5. Only *Bp*73 presents a fixed pattern of genotype (113/115) which could potentially be used as an identification marker for this strain (Additional file 1).

Within each population, *B. carambolae *Salaya5 strain is genetically comparable with *B. carambolae *Jakarta (the original strain) and Sumatra strains in terms of the number of alleles (*n*_a_), the effective number of alleles (*n*_e_), observed heterozygosity (*H*_O_), and expected heterozygosity (*H*_E_) (Table 1). In contrast, genetic variation of the Salaya5 strain is relatively higher than the Salaya1 strain for all four parameters.

**Table 1:** Genetic variation among the new genetic sexing Salaya5 strain, two parental strains, and two wild populations.

Sample	*n*_a_	*n*_e_	*H*_O_	*H*_E_	%*P*
*B. dorsalis *Salaya1	1.40	1.25	0.13	0.15	40.0
*B. dorsalis *Nakhon Pathom	11.60	6.01	0.58	0.81	100.0
*B. carambolae *Salaya5	3.40	2.17	0.43	0.41	80.0
*B. carambolae *Jakarta	4.40	2.21	0.35	0.43	80.0
*B. carambolae *Sumatra	2.40	1.70	0.21	0.36	100.0

n_a_, mean number of alleles; n_e_, mean effective number of alleles; H_O_, mean observed heterozygosity; H_E_, mean expected heterozygosity; %P, mean percentage of polymorphic loci.

**Figure 5:**
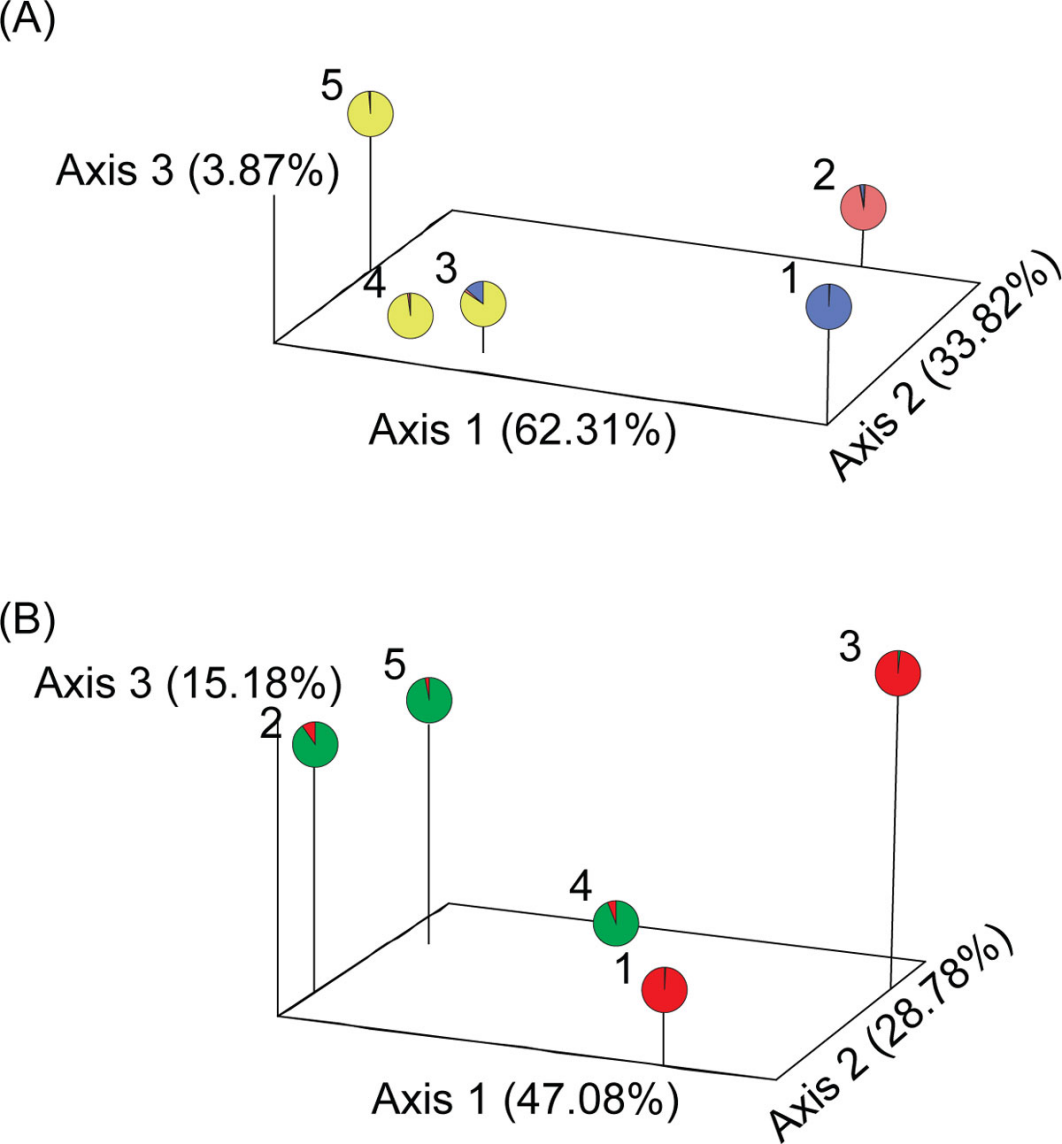
**Three-dimension plot of Principle Coordinate Analysis (PCoA) and STRUCTURE analyses**. The planes of the first three principle coordinates 62.31%, 33.82%, and 3.87% of total genetic variation, respectively, derived from five variable microsatellite loci (A). The planes of the first three principle coordinates 47.08%, 28.78%, and 15.18% of total genetic variation, respectively, derived from four Y-pseudo-linked microsatellite loci (B). The pie graph represents average membership distribution to three (A) and two (B) clusters, respectively. 1, *B. dorsalis *Salaya1 strain; 2, *B. dorsalis *wild strain; 3, *B. carambolae *Salaya5 strain; 4, *B. carambolae *Jakarta strain; 5, *B. carambolae *Sumatra strain.

Principle Coordinate Analysis (PCoA) derived from two different sets of microsatellite markers illustrates the genetic divergence of wild fruit fly populations and genetic sexing strains (Figure 5). Using five polymorphic microsatellite loci (Figure 5A), the first axis accounts for 62.31% of total variation and can distinguish two species (*B. dorsalis *and *B. carambolae*). The second (33.82% of total variation) mainly separates Salaya1 from wild *B. dorsalis *population, but still groups Salaya5 strain to *B. carambolae *Jakarta strain (the original strain) (Figure 5A). On the other hand, using other four Y-pseudo-linked microsatellite loci (Figure 5B), the first axis (47.08% of total variation) separates genetic sexing strains (Salaya1 and Salaya5) from the wild populations. The second axis, accounting for 28.78% of total variation, divides *B. dorsalis *from *B. carambolae*.

This inference is also supported by Bayesian cluster analysis (STRUCTURE) as shown in pie graphs of Figure 5. An optimal number of hypothetical cluster (*K*) was detected using Δ*K *[41]: *K *= 3 and *K *= 2 for five polymorphic and other four Y-pseudo-linked microsatellite loci, respectively. At *K *= 3, cluster 1 contains the Salaya5 strain (*Q *= 0.847) and two populations of *B. carambolae *(*Q*_Jakarta _= 0.978 and *Q*_Sumatra _= 0.988*)*. Additionally, the Salaya5 strain significantly shares the coancestry distribution (*Q *= 0.136) with *B. dorsalis *Salaya1 strain in cluster 3 (*Q *= 0.990) while *B. dorsalis *wild population (Nakhon pathom population) fits to cluster 2 (*Q *= 0.963). This subdivision is corresponded to the first and second axis of PCoA, respectively (Figure 5A). Likewise, using four Y-pseudo-linked microsatellite loci, at *K *= 2, two genetic sexing strains share the same cluster 2 (*Q*_Salaya1 _= 0.990 and *Q*_Salaya5 _= 0.982). The remaining three wild populations belong to cluster 1 (*Q*_Nakhon Pathom _= 0.905, *Q*_Jakarta _= 0.941, and *Q*_Sumatra _= 0.970). The result is congruent to the first principal axis of PCoA (Figure 5B). According to PCoA and STRUCTURE analyses, the Salaya5 strain carries most of the genome originated from *B. carambolae *Jakarta strain and sexing components from *B. dorsalis *Salaya1 strain.

The Salaya5 strain has a potential to be used in *B. carambolae *SIT programs based on male-only releases probably in wide geographical range because the sterile males can compete with the wild males from both Jakarta and Sumatra. However, further evaluations such as cytogenetic characterization, measures of productivity and the stability of genetic sexing characters under mass-rearing conditions, and open field trial would be required to better state the suitability of the Salaya5 strain for SIT. The five polymorphic microsatellite markers (*Bd*1, *Bd*9, *Bp*125, *Bp*173, and *Bp*181) can be used to evaluate the maintenance of genetic variability in Salaya5 under mass-rearing conditions. In addition, a Y-pseudo-linked microsatellite marker genotype was also fixed in the Salaya5 (Additional file 1). Therefore, the utilization of this marker for strain identification in field monitoring traps is also possible as in the case of the Salaya1 [30]. In addition, this type of the Y-pseudo-linked marker provides an opportunity to further develop sperm marking for the study of mating performance of sterile males if they are found to be unique characters [42,43].

## Conclusions

We report the first successful example of the construction of the Salaya5 GSS for SIT by introducing genetic sexing components developed in one species, *Bactrocera dorsalis *Salaya1 strain into the genome of another species, a closely related *B. carambolae *via introgression. A range of evidence such as morphology-based taxonomic characteristics, distinctive pheromone component, microsatellite DNA markers, genetic relationships, and mating competitiveness provided parental baseline data and validation tools for the new strain. The Salaya5 strain shows much closer similarity of those features toward the wild *B. carambolae*. The results of mating competitiveness revealed that Salaya5 GSS is comparable to wild males in mating with wild females. The Salaya5 strain has the potential to be used in *B. carambolae *SIT programs based on male-only releases although further evaluation at the mass-rearing conditions and field trial levels would be required to authenticate the practical application of the Salaya5 for SIT programs.

## Methods

### Fruit fly sources

The Salaya1 strain, a Y-autosome translocation based genetic sexing strain of *B. dorsalis *based on a brown-white pupal color dimorphism, was reared in a laboratory cage with the dimensions of 0.35 × 0.45 × 0.35 m (width × length × height) under a photoperiod of 13:11 [L:D] h at 25-28 °C with 70-80% RH. Adult flies were provided a mixture of yeast hydrolysate and sugar (1:3). The wild *B. dorsalis *population samples were collected from mango hosts, *Mangifera indica*, from Nakhon Pathom, Thailand, while the wild *B. carambolae *individuals were obtained from carambola fruit hosts, *Averrhoa carambola *L., collected from Jakarta and Sumatra, Indonesia. These infested fruits were separated and sorted for wild fruit fly larvae. Approximately 600 flies were used to establish colonies. The rearing conditions were the same as for the Salaya1 strain.

### Introgression of two principle components for genetic sexing strain into the genetic background and gene pool of *B. carambolae*

The Salaya1 is genetic sexing strain based on a translocation and a brown-white pupal color dimorphism [30]. It contains two principle components: a free autosome carrying white pupae mutant allele (A*^wp^*) and a Y-autosome translocation that links the dominant wild-type allele of the white pupae (Y-A*^wp+^*). The males are heterozygous (Y-A*^wp+^*/A*^wp^*) displaying a brown pupae phenotype while the females are homozygous recessive (A*^wp^*/A*^wp^*) for white pupae phenotype and can be distinguished from the males.

The two principle components were initially introduced into *B. carambolae *genetic background by an interspecific cross between parental (P) males of the Salaya1 strain and parental (P) virgin females of the *B. carambolae *in mass (50 males × 50 females) from Jakarta (Figure 4). All of the resulting F_1 _progeny, having 50% genetic background of *B. carambolae*, were interspecific hybrids with the brown pupae phenotype. The female (X/X) hybrids were A*^wp+^*/A*^wp ^*heterozygotes while the male hybrids were homozygotes carrying Y-A*^wp+^*/X; A-Y/A*^wp+^*. Subsequently, males (B2M) and virgin females (B2F) hybrid lines were repeatedly backcrossed in mass (50 males × 50 females) of the respective virgin females and males of *B. carambolae*.

In case of the female hybrid line (B2F), the F_1 _females (with A*^wp+^*/A*^wp ^*genotype) were backcrossed with the males of *B. carambolae *(with A*^wp+^*/A*^wp+^*genotype) in mass (50 females × 50 males). The resulting F_2 _progeny, having already 75% genetic background of *B. carambolae*, were all brown pupae and not selectable for the recessive A*^wp^*. It was necessary to inbreed the F_2 _progeny in order to select the white pupae female individuals (A*^wp^*/A*^wp^*) in the F_3 _generation. The selected white pupae females were then ready for the other rounds of backcrosses. All of the F_4 _female progenies were again heterozygotes, (A*^wp+^*/A*^wp^*), as the F_1 _generation. However, they conceptually constituted 87.5% of the *B. carambolae *genetic background. The F_4 _generation females were ready to follow other rounds of backcrosses and inbreeding as indicated for the F_1 _to F_3 _generations in the left handed column of Figure 4. Alternation of these crosses continued until the F_12 _generation.

For the male hybrid line (B2M), the F_1 _males (with Y-A*^wp+^*;A*^wp+^*) were back crossed with virgin females of the *B. carambolae *(with A*^wp+^*/A*^wp+^*) in mass (50 males × 50 females) as shown in the right column of Figure 4. All of the resulting F_2 _progenies were brown pupae and all males were carrying the Y-A*^wp+^*component. Therefore, 50 male progenies could be directly selected and repeatedly backcrossed to 50 virgin females of the *B. carambolae*. The same backcrosses were carried out until the F_10 _generation. Subsequently, virgin (only white pupae) females and males from the B2F and B2M lines, respectively, were crossed in mass (50 females × 50 males). The resulting brown pupae male progenies of these crosses were heterozygous for the pupal color gene, carrying Y-A*^wp+^*/X;A-Y/A*^wp ^*as the Salaya1 males (Figure 4; bottom middle column). These heterozygote brown pupae males were finally crossed with the white pupae B2F females (A*^wp^*/A*^wp^*) in mass (50 males × 50 females) to establish a true breeding genetic sexing strain that had brown-white pupae color sexual dimorphisms in the *B. carambolae *genetic background.

### Morphological characterization

A taxonomic report by Drew and Hancock [7] was used for reference regarding morphological discrimination of fruit flies. *B. carambolae *can be distinguished from *B. dorsalis *by three key traits: (1) the abdominal terga III-V, the medial longitudinal band, and the anterolateral corners of tergum IV; (2) the legs (femora); and (3) the wing costal band.

### Male pheromonal component analysis

Sexually mature (20-28 days old) specimens of parental *B. carambolae *(Jakarta) and *B. dorsalis *Salaya1 strain were used for analysis of pheromone profile from the rectal glands. Fifteen to twenty males were allowed to feed on 5 μl of methyl eugenol (ME); dispensed on a small piece of filter paper (2 × 2 cm^2^; Whatman^⊗ ^No. 1) for about 15 min during their peak response to ME (10:00-11:00) [16]. After 24 hours, the rectal glands were individually dissected and placed in a screw-cap glass vial containing 50-100 μl of absolute ethanol and stored at -20 °C for further analysis. Each rectal gland was homogenized by 200 micropipette tip shearing and followed by a 5 min vortexing. The extract was transferred into a new microtube and dried up by adding granular sodium sulphate anhydrous. After a centrifugation at 13,000 rpm for 5 min, the supernatant was collected in a conical glass inserted inside a screw-cap glass vial for gas chromatography-mass spectrometry (GC-MS) analysis.

All rectal gland extracts were tested using a GC-MS system [GC: Agilent 6890] with an HP-5MS capillary column (30 m, 0.25 mm i.d., 0.25 μm film thickness); MS: mass selective detector 5973N with an ionization energy of 70 eV. The carrier gas was helium with flow rate 1 ml/min. Column temperature was initially set at 40 °C, then gradually increased to 240 °C at 5 °C/min, and held for 5 min. One-microliter aliquot of the extract was automatically injected via spilt mode (10:1) with injection temperature of 220 to 270 °C. Identification of pheromone components was based on computer matching with the Wiley7n.L mass spectral library, as well as comparisons of the fragmentation pattern of the mass spectra with data published [20].

### Mating competitiveness in field cages

Wild (F_1 _to F_2_) *B. carambolae *flies were sex separated within 24 hours after eclosion and held in cages with a maximum density of 40 flies per liter. The tested Salaya1 or Salaya5 brown pupae were irradiated with gamma rays from a cobalt-60 source at a dose of 50 Gy during their late pupal stage (two days before emergence) and irradiated males were held in the same manner as untreated wild flies. The sexual maturity and mating time of each strain were ensured by observing the displayed courtship behaviour in the insectary. Mating experiments were carried out according to the standard protocol which includes periodic quality control tests from FAO/IAEA/USDA [44]. A mating competitiveness study was conducted in outdoor field cages (3.0 m width × 3.5 m length × 2.3 m height) having a potted mango tree inside.

The field cage testing constituted a wild control cage into which 20 pairs of sexually mature carambola fruit flies were released. Secondly, an experimental competitive mating cage had the same 20 pairs of the untreated wild flies and the other 20 sterile tested males (Salaya1 or Salaya5 strains) were released. In this case, a dot of water-based color was painted on the scutum of the individual sterile males using a soft paint brush (for at least 48 hours before the field cage testing) for future discrimination of male strains. Number of copulation obtained from each possible mating combination (*B. carambolae *♂ × *B. carambolae *♀ and any tested sterile ♂ × *B. carambolae *♀) was recorded from field cage test. The data for the propensity of mating (PM) was calculated, which reflects whether the developmental condition is satisfactory for mating activity. The PM is considered adequate when 50% of all mating combinations occur. If the results are less than 20% of any combination, this data should be discarded. The relative sterile index (RSI) is a major index of male sexual competitiveness. The values of RSI range from 0 (all wild females mate with wild males) to 1 (all of wild females mate with sterile males). A value of 0.5 indicates an equal mating performance for wild and sterile males. In addition, mated females were collected and held in a cage. They were exposed to an egger (punctured plastic vial) coated inside with guava juice as an oviposition stimulant. The eggs were subsequently transferred onto moist paper with artificial larval food in order to assess the egg hatching rate. This data was used to calculate the Fried's competitiveness coefficient (C), which is an index of overall mating competitiveness of sterile males. The C value indicates if sterile males from developed strains are less or equally competitive than the target wild males. The C value ranges from 0 (better competitiveness of wild males) to 1 (equal competitiveness between sterile and wild males). All tests were repeated three times. A *t*-test was used to compare the mean competitiveness index among mating experiments using PASW statistics software v18.0 (SPSS).

### Genomic DNA extraction, microsatellite DNA amplification and genotyping

Thirty male individuals of each population (*B. carambolae *(Jakarta), *B. carambolae *(Sumatra), *B. dorsalis *(Nakhon Pathom), the Salaya1 colony, and the resulting introgressive strain (Salaya5)) were used for genotyping. The genomic DNA was extracted from each individual fly as described by Aketarawong and colleagues [45]. PCR amplifications using two sets of microsatellite loci; five polymorphic loci (*Bd*1, *Bd*9, *Bp*125, *Bp*173, and *Bp*181) and four Y-pseudo-linked markers (*Bd*15, *Bd*42, *Bp*58, and *Bp*73) [30] were carried out in order to assess general genetic background and validate the existence of the Y- A*^wp+ ^*component in the Salaya5 strain, respectively. PCR reactions and conditions were performed using the thermal cycler Flexcycler (Analytik Jena AG, Jena, Germany). PCR products were run on 6% or 12% polyacrylamide gels and were scored in comparison with a 25 bp DNA ladder (Promega, Madison, WI, USA) as described in [13].

### Population genetic analyses

Genetic variations (i.e., *n*_a_, *n*_e_, *H*_o_, and *H*_E_) of five variable microsatellite loci (*Bd*1, *Bd*9, *Bp*125, *Bp*173, and *Bp*181) were measured using GENALEX v.6.5 [46]. Departure from Hardy-Weinberg equilibrium and linkage disequilibrium was determined using GENEPOP v.4 [47], with their critical levels after the sequential Bonferroni test [40]. For four Y-pseudo-linked microsatellite markers (*Bd*15, *Bd*42, *Bp*53, and *Bp*73), allele and genotypic frequencies were calculated in order to define a potential marker for strain identification.

The Principle Coordinate Analysis (PCoA) performed on genetic distance was analyzed for displaying genetic divergence among the individual samples in multidimensional space, using the GENALEX v.6.5 [46]. The first three principal coordinates were plotted using the subprogram MOD3D in NYSYS-pc v.2.1 [48]. To identify the number of potential genetic cluster (*K*), the Bayesian approach implemented in the program STRUCTURE v.2.3.2 [49,50] was used. The program was run for *K *= 1 to *K *= 5, using the admixture model with correlated allele frequencies and default parameters: prior *F*_ST _mean of 0.01, different values of *F*_ST _for different subpopulations, and a standard deviation of 0.05. All runs were repeated 10 times with the condition of the burn-in period 100,000 steps and 500,000 MCMC repetitions. The most likely genetic cluster was determined by the Delta *K *method [41].

## Competing interests

The authors declare that they have no competing interests.

## Authors' contributions

SI and ST participated in design of a research project. SI, NA, SL, and ST performed experiments and data analyses. SI, NA, and ST drafted the manuscript. All authors reviewed and approved the final manuscript.

## Supplementary Material

Additional file 1 **Table S1**. Genotypic frequency of four Y-pseudo-linked microsatellite loci in each population. Established strain identification markers in the Salaya1 strain [30] are in bold. A potential strain identification marker in the new genetic sexing strain, Salaya5 is underlined. Click here for file
