# Supplementary material for: Development of a genetic sexing strain in Bactrocera carambolae (Diptera: Tephritidae) by introgression of sex sorting components from B. dorsalis, Salaya1 strain
Source: BMC Genet. 2014 Dec 1;15(Suppl 2):S2. doi: 10.1186/1471-2156-15-S2-S2 (PMC4255791; doi:10.1186/1471-2156-15-S2-S2)
Supplement: Additional file 1 — Table S1. Genotypic frequency of four Y-pseudo-linked microsatellite loci in each population. Established strain identification markers in the Salaya1 strain [30] are in bold. A potential strain identification marker in the new genetic sexing strain, Salaya5 is underlined. [file 1471-2156-15-S2-S2-S1.docx]

**Table S1** Genotypic frequency of four Y-pseudo-linked microsatellite loci in each population. Established strain identification markers in the Salaya1 strain [30] are in bold. A potential strain identification marker in the new genetic sexing strain, Salaya5 is underlined.

|  |  | *B. dorsalis* | |  | *B. carambolae* | | |
| --- | --- | --- | --- | --- | --- | --- | --- |
| Locus | Genotyoe | Salaya1 | Nakhon Pathom |  | Salaya5 | Jakarta | Sumatra |
| *Bd*15 | 191/194 | 0.000 | 0.000 |  | 0.871 | 0.000 | 0.000 |
|  | 191/196 | 0.000 | 0.000 |  | 0.097 | 0.000 | 0.000 |
|  | **191/192** | 1.000 | 0.000 |  | 0.032 | 0.000 | 0.000 |
|  | Other genotypes | 0.000 | 1.000 |  | 0.000 | 1.000 | 1.000 |
|  |  |  |  |  |  |  |  |
| *Bd*42 | **181/186** | 0.467 | 0.000 |  | 0.065 | 0.000 | 0.000 |
|  | 185/186 | 0.000 | 0.000 |  | 0.097 | 0.000 | 0.000 |
|  | **186/186** | 0.533 | 0.000 |  | 0.839 | 0.167 | 0.469 |
|  | Other genotypes | 0.000 | 1.000 |  | 0.000 | 0.833 | 0.531 |
|  |  |  |  |  |  |  |  |
| *Bp*58 | **116/118** | 1.000 | 0.000 |  | 0.097 | 0.033 | 0.125 |
|  | 118/118 | 0.000 | 0.114 |  | 0.871 | 0.200 | 0.250 |
|  | 118/121 | 0.000 | 0.000 |  | 0.032 | 0.000 | 0.250 |
|  | Other genotypes | 1.000 | 0.885 |  | 0.000 | 0.767 | 0.375 |
|  |  |  |  |  |  |  |  |
| *Bp*73 | **111/113** | 1.000 | 0.086 |  | 0.000 | 0.000 | 0.000 |
|  | 113/115 | 0.000 | 0.114 |  | 1.000 | 0.000 | 0.000 |
|  | Other genotypes | 0.000 | 0.800 |  | 0.000 | 1.000 | 1.000 |
